# Pulmonary Nodule Clinical Trial Data Collection and Intelligent Differential Diagnosis for Medical Internet of Things

**DOI:** 10.1155/2022/2058284

**Published:** 2022-05-26

**Authors:** Weijia Wu, Lizhong Gu, Yuefeng Zhang, Xianping Huang, Weihe Zhou

**Affiliations:** Department of Cardiothoracic Surgery, The Second Affiliated Hospital of Wenzhou Medical University, Wenzhou, Zhejiang 325000, China

## Abstract

In this paper, the medical Internet of things (IoT) is used to pool data from clinical trials of pulmonary nodules, and on this basis, intelligent differential diagnosis techniques are investigated. A filtered orthogonal frequency division multiplexing model based on polarisation coding is proposed, where the input data are fed to a modulator after polarisation cascade coding, and the system performance is analysed under a medical Internet of things modulated additive Gaussian white noise channel. The above polarisation-coded filtered orthogonal frequency division multiplexing system components are applied to electroencephalogram (EEG) signal transmission, to which a threshold compression module and a vector reconstruction module are added to address the system power burden associated with the acquisition and transmission of large amounts of real-time EEG data in the medical IoT. In the threshold compression module, the inherent characteristics of EEG signals are analysed, and the generated EEG data are decomposed into multiple symbolic streams and compressed by applying different thresholds to improve the compression ratio while ensuring the quality of service of the application. A deep neural network-based approach is proposed for the detection and diagnosis of lung nodules. Automatic identification and measurement of simulated lung nodules and the corresponding volumes of nodules in images under different conditions are applied. The sensitivity of each AIADS in identifying lung nodules under different convolution kernel conditions, false positives (FP), false negatives (FN), relative volume errors (RVE), the miss detection rate (MDR) for different types of lung nodules, and the performance of each system in predicting the four types of nodules are calculated. In this paper, an interpretable multibranch feature convolutional neural network model is proposed for the diagnosis of benign and malignant lung nodules. It is demonstrated that the proposed model not only yields interpretable lung nodule classification results but also achieves better lung nodule classification performance with an accuracy rate of 97.8%.

## 1. Introduction

Relevant literature shows that pulmonary nodules may be transformed into lung cancer, so the early diagnosis of pulmonary nodules is very important for patients. Lung cancer is not formed in one day, which needs our attention [1]. Before it forms a tumor, it often appears as small pulmonary nodules. A pulmonary nodule is only a pathological localized lesion of the lung. Generally, it can be inferred whether it will develop into a malignant tumor according to the size, shape, and growth rate of the nodule [2]. Only when lung cancer is detected in the early stage can it increase the relative survival rate and survival rate [3]. Therefore, the detection of pulmonary nodules is very important for the screening of lung cancer, which can increase the probability of finding early cancer cells in asymptomatic lung cancer patients. The main manifestation of lung cancer is abnormal cells with rapidly increasing growth rate, forming a mass called pulmonary nodule in the lung [4]. As an early manifestation of lung cancer, pulmonary nodules have a complex relationship with cancer [5]. The existence of pulmonary nodules does not necessarily mean cancer, but it is still necessary to accurately and carefully analyze each suspicious nodule in order to better provide an effective method for early diagnosis of lung cancer.

Pulmonary nodules have the characteristics of small volume, complex shape, difficult early detection, and easy to be mistaken for adjacent organs, so its detection and diagnosis are a challenging task [6]. However, the scanning speed and imaging quantity of lung CT images increase, which makes manual detection time-consuming and brings a great workload to radiologists [7]. At the same time, the lesions in CT images may have the characteristics of insignificant performance or diverse changes, which makes the detection difficult. Doctors need to have rich experience and knowledge. It is easy to miss diagnosis and misdiagnosis only by doctors' naked eye observation, which will affect the detection results and later treatment. Computer-aided detection (CAD) system uses computer-related technology, combined with medical image processing and other means, which can assist doctors to diagnose diseases efficiently [8]. In the task of pulmonary nodule detection, assisting doctors to detect lung lesions can effectively improve the efficiency and accuracy of pulmonary nodule detection. With the rapid development of computer hardware, software, and other technologies, computers gradually have powerful storage and computing capabilities to process massive data. Therefore, excellent algorithms based on machine learning (ML) and deep learning (DL) have emerged to solve the problem of pulmonary nodule detection, so as to better help doctors improve diagnostic efficiency and further improve the survival rate of patients [9–11].

**Figure 1:**
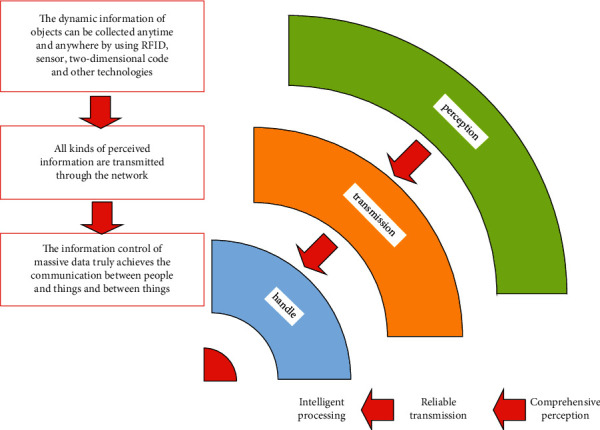
Characteristics of the Internet of things.

The Internet of things is a new type of network based on the Internet, telecommunication network, and other information. It can interconnect all ordinary physical objects that can be addressed independently. As shown in Figure 1, the characteristics of the Internet of things are obvious. First, the Internet of things can provide the connection between sensors, and then, the Internet of things can intelligently process and control objects. We combine sensors and intelligent processing through the Internet of things and then use various intelligent technologies such as cloud computing and pattern recognition to expand relevant fields [12–15]. The Internet of things makes full use of new technologies, such as radio frequency identification technology, sensor technology, and nanotechnology in all walks of life, fully connects all kinds of objects, and sends all kinds of real-time dynamic information collected to the computing and processing center through wireless network for summary, analysis, and processing [13, 16, 17]. Computers can be used to centrally manage and control machines, equipment, and personnel, optimize production and life in a more refined and dynamic way, and realize the organic integration and harmonious coexistence of human society and the material world [15, 18–20].

## 2. Related Work

Health and medical Internet of things is an important application field of Internet of things technology in the medical industry [21]. In 2018, China clearly proposed to “actively connect with the medical consortium, use Internet technology to speed up the exchange and sharing of medical resources and information, achieve efficient business coordination, and carry out more convenient telemedicine services” [22]. At present, the medical-related Internet of things can be roughly divided into three application fields: smart hospital service, home health service, and public health service, covering multiple subapplications such as drug traceability, intensive care, medical consumables management, and health management. With the increase in people's desire for a better life, people's demand for home health services has gradually increased, and the Internet of things platform for home health services has also been paid full attention [23].

However, most of these applications still rely on regional developed medical resources to do some support, and the use is also very complex. In areas with backward medical levels, the utilization rate of Internet of things medical services is not high [24]. At the same time, the user's personal data involve personal privacy and interests. Once it is leaked, it will have a serious impact on users [25]. Most of the current medical Internet of things products do not consider the problem of data security, and many will even transfer the user's data directly to the cloud disk, which has great hidden dangers. Therefore, data security in smart medicine has also been the focus of attention of governments and researchers in various countries since 2020. Most of the traditional lung nodule detection methods are manually designed to extract features. The general process is lung parenchyma segmentation, candidate region extraction, and reducing false-positive classification. Based on this, Yan et al. [26] used a three-dimensional adaptive fuzzy threshold and morphological hole-filling method to obtain a fine lung mask. Then, 15 relevant features of pulmonary nodules are extracted, including intensity-based features, random features (skewness and kurtosis), and shape index. A support vector classifier is used to remove the nonnodule part and reduce the number of false positives. Prabukumar et al. [27] detected candidate nodules, and they continuously used two k-nearest neighbor classifiers to determine the number of false-positive nodules. Lv et al. [28] segmented pulmonary nodules and blood vessels by using the enhancement filter method and then located the divergence feature of the central cluster of nodules. In the false-positive classification stage, genetic algorithm and artificial neural network are used to select features to get the detection results.

Kim et al. [29] used an adaptive Wiener filter to optimize the image. After preprocessing, they used the edge search method for fast lung segmentation. Zhou et al. [30] first segmented the lung parenchymal region by modeling, transferred the nonsolitary nodule to the solitary nodule by mask technology, and used the support vector algorithm to locate the nodule by fully combining the anatomical and random features of the nodule. The total detection accuracy of the algorithm for real, nonreal, and hollow nodules is 89%. The above traditional lung nodule detection algorithm is complex and cannot achieve end-to-end detection. Manual feature extraction requires rich medical expertise to extract effective features. In addition, manual feature selection is difficult to have good robustness in practical application.

Most of the algorithms are only for modeling some types of nodules, which is far from practical application. Wang et al. [31] applied transfer learning to extract the discriminant features of three-dimensional lung nodules and introduced multitask learning for classification. Cheng et al. [32] proposed an interpretable hierarchical semantic convolution neural network, which quantifies the diagnostic features used by radiologists through low-level semantic output and explains how the model interprets images in an expert manner. Ueda et al. [33] selected four groups of stereo data with different slice numbers from the stereo data containing nodules as the initial features, used the U-net network structure to extract the features and compare the classification results, and added the feature visualization technology to prove the effectiveness and interpretability of the features.

## 3. Construction and Key Technologies of Medical Internet of Things

### 3.1. Architecture of the Internet of Things

**Figure 2:**
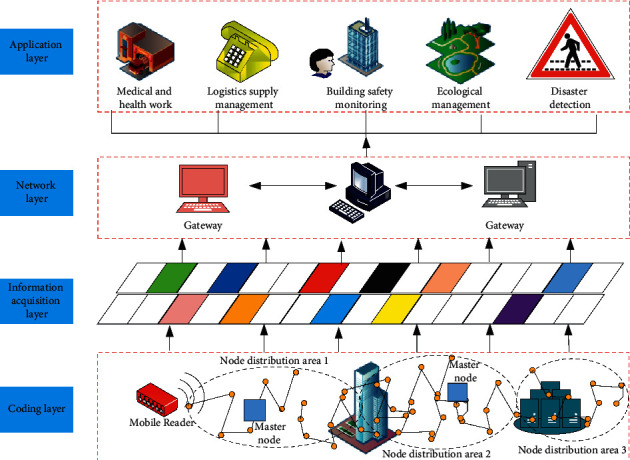
Architecture of Internet of things.

The Internet of things is mainly divided into four layers: coding layer, acquisition layer, network technology layer, and application technology layer, as shown in Figure 2. With the rapid development of hardware computing power, algorithms, and data, deep learning has moved from the research field to the practical application level in medicine and has been applied to assist in diagnosis and treatment, disease risk prediction, and big data health analysis. Deep learning has two features, one of which is high computational efficiency and the other is the ability to give accurate data analysis and decision making, which can fundamentally solve the pain point of oversupply of medical services.

The coding layer is the digital name of goods, equipment, location, and attributes. The information acquisition layer refers to the process of obtaining article coding information through automatic identification and short-range communication technologies including bar code, radio frequency identification, Bluetooth, and wireless sensor. The network layer is the communication network for information exchange, including wireless communication networks such as WiFi. The lung nodule intelligent assisted diagnosis system applies deep learning and other algorithms to the field of medical image analysis and processing, using computers to detect and diagnose lung nodules on chest CT images, and using the diagnosis and labelling results given by computers as reference suggestions for radiologists' clinical diagnosis, which can relieve the work pressure and burden of radiologists and improve the accuracy and efficiency of early lung cancer screening.

**Figure 3:**
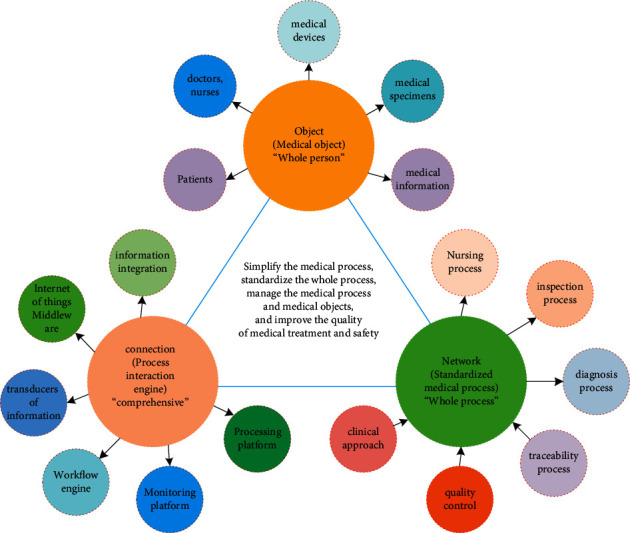
Core concept of medical Internet of things.

Based on the above needs, combined with the good performance of the lung nodule detection and diagnosis algorithm above, a deep learning-based intelligent assisted diagnosis system for lung nodules is designed and implemented, which is demanded by radiologists. The system can store chest CT image data, view relevant personal information of patients and their CT image sequences, and then automatically detect and diagnose lung nodules and visualize them through the interface All the doctor needs to do is to review and modify the computerized results to reduce computer misdiagnosis. The application layer is an application system built on the technical architecture of the Internet of things, including different application systems such as commercial trade, coordination, agriculture, medical treatment, and military. The Internet of things is a very complex and diverse system technology. According to different application fields and different information collection and processing methods, the hierarchical division methods are also different. The core concept of the medical Internet of things is shown in Figure 3.

### 3.2. Key Technologies of Medical Internet

#### 3.2.1. Wireless Sensor Technology

**Figure 4:**
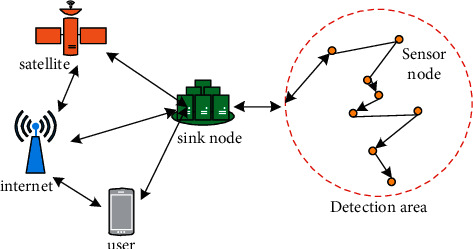
WSN plane topology.

A wireless sensor network (WSN) is a multihop self-organizing network system formed by many sensor nodes through wireless communication. Traditional wireless networks can improve the quality of service, the effective utilization of bandwidth, and save energy. In the beginning, wireless sensor design is to make effective use of energy, which is also one of the most important differences between new wireless sensor networks and traditional old networks. The plane topology of WSN is shown in Figure 4.

#### 3.2.2. RFID Technology

The emergence of RFID technology has greatly enriched the connotation of Internet of things technology. Generally speaking, RFID technology is a system that combines network technology and database technology. In the Internet of things technology, RFID technology can collect central information and identify commodity information through a wireless data communication network automatic acquisition system. Then, information exchange and sharing are realized through the computer network to realize the flat management of goods. RFID technology originates from radar technology, and its working principle is similar to radar. First, the card reader sends an electronic signal. After receiving the signal, the tag sends internal relevant information. Then, the reader sends the identified tag information back. Finally, the reader sends the recognition result to the host.

## 4. Data Collection and Monitoring of Pulmonary Nodules Based on Medical Internet of Things

### 4.1. Data Collection and Monitoring Methods of Pulmonary Nodules

**Figure 5:**
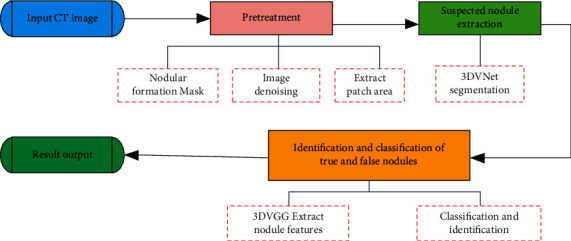
Overall algorithm of pulmonary nodule detection and recognition.

This chapter collects and diagnoses the clinical data of pulmonary nodules based on the Internet of things, which is mainly divided into the model algorithm part and data set evaluation part. First, the algorithm flow of pulmonary nodule detection and recognition is introduced, and the CT images of the data set are preprocessed. Then, the UNET network model of lung nodule detection in the algorithm part of the model is introduced, and the 3DVNet network deformed by the UNET network is used for image cutting. Then, the data set evaluation part is introduced, the generated cubes are resampled and data-enhanced, and the generated samples are used as the input of the 3DVNet network to train the detection model. Finally, the effects of two data enhancement methods based on a three-dimensional pixel statistical generation algorithm and generation countermeasure network on lung nodule recognition of the 3DVNet model are compared, and the improvement of lung nodule model detection effect by introducing a hidden variable embedding mechanism into the network is explored. The whole process of pulmonary nodule detection and recognition algorithm is divided into the following steps: (1) preprocessing CT images; (2) extraction of suspected nodules; (3) identification of true and false nodules. The algorithm flow is shown in Figure 5.

Medical image data are often very large, and the hardware equipment is not enough to support network training. Therefore, it is necessary to slice the image before it can be sent to deep network training, but there will be many overlapping parts and redundant operations.

The intelligent diagnostic results are presented to the physician in the form of a table covering the diagnostic details for each slice with a nodule in the CT image sequence of the case. The diagnostic details include the CT image layer number, the coordinate information of the lung nodule (width and height of the outer rectangle of the contour), the probability of the malignancy of the lung nodule, and the probability of the corresponding semantic features (burr sign, lobar sign, and contrast); at the same time, the doctor can swipe the mouse or click on a row in the table to see the visualization of the corresponding nodule, with the yellow outline on the image indicating the identified and localized lung nodule, next to the corresponding diagnostic description.

If we want to obtain more spatial context information as much as possible, we will sacrifice the positioning accuracy. UNet is a classical image segmentation network model. This simple structure can reduce the problem of network training and can still obtain better segmentation accuracy even when there are few data sets.

UNet model has many advantages: the first advantage comes from its structure–encoding and decoding structure, which can obtain the characteristics of the image at the same time, restore it well, and get very accurate results. The second advantage is that even if there are few training samples, it will not affect the segmentation task too much, and the efficiency is higher than that in other models. Third, the end-to-end connection can directly process the complete image, complete the segmentation task, and retain all the information of the input image. Compared with the patch-based segmentation method, this is a very prominent advantage. The most common areas of using 3D images are medical 3D scanning and video processing. When processing 3D data, information in three dimensions (video timeline) will be used. When lung oncologists mark lung nodules in human lung scans, they look at adjacent image slices to determine whether the part is part of the lesion. In computer vision, the two-dimensional form is replaced by the three-dimensional form with higher accuracy. There are two methods for 3D image transmission learning. The first is to train the 3D network, which is designed for migration learning on the existing 3D data set. The second is to use the existing 2D network and convert it into 3D processing.

Deep neural networks require a large amount of data for learning. In this paper, only the LUNA2016 dataset and the LIDC-IDRI dataset were used, which is not enough data. Although data augmentation techniques were used to expand the data volume, the data samples are still insufficient for deep learning. In future research work, a large-scale lung CT image dataset can be constructed or clinical image data can be used to improve the performance of nodule detection and diagnosis.

**Figure 6:**
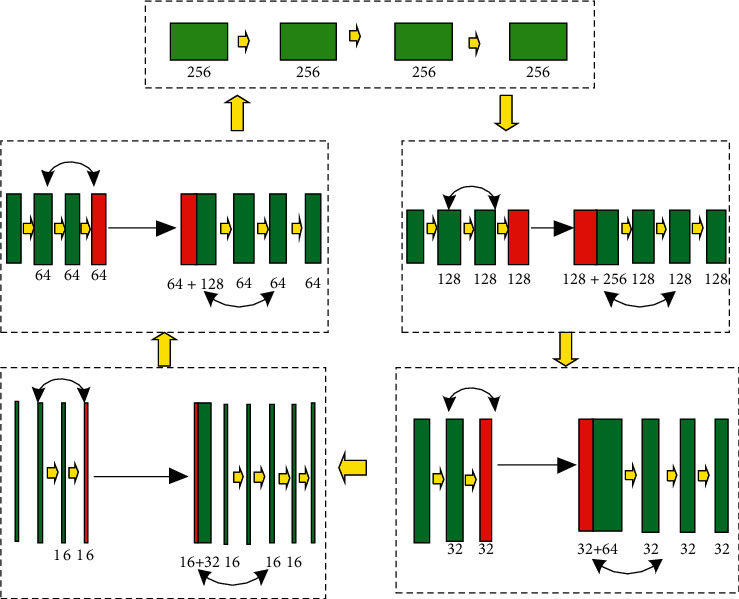
Schematic diagram of 3DVNet network structure.

In this paper, the 3DVNet network is used to complete the task of medical image segmentation. 3DVNet is a deep learning method based on UNet. The residual block is added by using the network structure of UNet. The network is derived from 2D to 3D. 3DVNet is very suitable for 3D medical image processing. In this paper, the 3DVNet network is used to complete the task of medical image segmentation. 3DVNet is a deep learning method based on UNet. The residual block is added by using the network structure of UNet. The network is derived from 2D to 3D. 3DVNet is very suitable for 3D medical image processing. In the segmentation problem, the large receiving field provides context information, while the small receiving field provides fine-grained information, which helps to improve the accuracy of segmentation.  Figure 6 shows a schematic diagram of the 3DVNet network structure.

### 4.2. Experimental Results and Analysis

#### 4.2.1. Influence of Data Enhancement Based on Generative Model on Model Effect

In this paper, some semantic features and image features learned by deep neural networks are used for the detection and diagnosis of lung CT images. In practical clinical diagnosis, the diagnosis of lung nodules is also closely related to factors such as the patient's smoking history, family case history, patient's age, and work environment, and future research work could improve the performance of the model if more comprehensive feature information is incorporated into the model.

**Figure 7:**
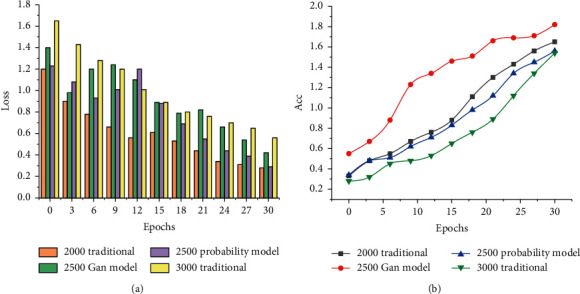
Accuracy and loss curve. (a) Loss histogram. (b) Accuracy curve.

To better show the training improvement results of the classification test data set on the 3DVNet model, the accuracy of the training set and the predicted graph are compared and analysed. The accuracy and loss function curves of the 3DVNet model trained with different test sets are shown in Figure 7.

At present, deep learning models for the diagnosis of benign and malignant lung nodules have problems of low interpretability and low practicability. To this end, this paper proposes an interpretable multibranch convolutional neural network model for the diagnosis of benign and malignant pulmonary nodules. In this paper, semantic features with high sensitivity to benign and malignant pulmonary nodules are selected and fused with a convolutional neural network to classify pulmonary nodules to form a multibranch network. Each branch extracts different characteristics of pulmonary nodules, which can not only predict the characteristics of pulmonary nodules but also diagnose the degree of malignancy, so that the diagnosis results are interpretable and provide a diagnosis basis for doctors.

2000 traditional represents dataset 1 (LUNA16 dataset 2000); 3000 traditional represents dataset 2 (LUNA16 dataset 3000); 2500GAN model means generating model data set 3 by generating countermeasure network method (LUNA16 data set 2000 + GAN generation 500); 2500 probability model represents the lung nodule generation algorithm of three-dimensional pixel statistics to generate data set 4 (LUNA16 data set 2000 + probability generation 500).

#### 4.2.2. Improvement Effect of Model Structure Based on Implicit Variable Embedding Mechanism

**Figure 8:**
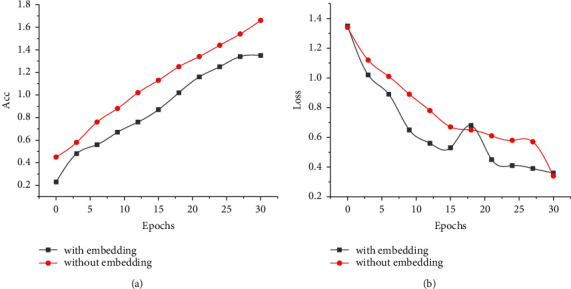
Model training accuracy and loss curve. (a) Loss curve. (b) Accuracy curve.

According to the previous work, this paper uses the implicit variable embedding mechanism to test in the Mnist database of handwritten digits. The Mnist test data set with noise is used to perform the classification task, and the implicit variable embedding mechanism is introduced into the classification network. The results show that the network with an embedding mechanism is better than the traditional network in the image classification task with noise, so the network with an embedding mechanism has more noise resistance. Therefore, this paper applies pixel embedding technology to any detection and recognition task of pulmonary nodules to analyze the robustness and training effect of the model. The experimental data set is trained by data set 1 (LUNA16 data set 2000) with an implicit variable embedding mechanism and compared with the training results of previous data set 1. The comparison results of embedding mechanism models are shown in Figure 8.

It can be seen from Figure 8 that in the initial stage of model training by adding an implicit variable embedding mechanism, the loss curve and accuracy curve show a stable trend with the increase in training times. Using the embedding mechanism in the 3DVNet network can improve the accuracy of the network, and the accuracy is improved by 1.6%. This section preliminarily proves from the experimental point of view that introducing an embedding mechanism into the network model of lung nodule detection can improve the accuracy of model training. The test set is derived from 4 cases in LUNA16 and selected 619 positive samples and 7233 negative samples. In order to balance the data set, 60 positive samples and 60 negative samples were randomly selected for experiments. When testing the model, it is found that the prediction Mask map obtained by the test results is also very poor, and the accuracy rate is low. According to the results of the training set, there is overfitting in the training process, which may be due to the lack of sufficient training data. Only 11 of the 818 cases in the LUNA16 data set are selected as the experimental samples, and their full data are not used.

To address the problems of complex and inefficient lung nodule detection and many false-positive results, this paper proposes an end-to-end 2D U-net combined with 3D CNN for lung nodule detection, in which 2D U-net is used for object localization and 3D CNN is used for stereoscopic target classification. The 2D U-net was first used for the initial detection of CT images to quickly identify and locate suspected nodules in the CT images, then the 3D image cubes of the suspected nodules were truncated according to the coordinate values of the suspected nodules and input to the 3D CNN model for training, and finally, the trained 3D model was used for binary classification of the suspected nodules to remove false-positive nodules. Experiments were conducted on the LUNA2016 and LIDC-IDRI datasets to demonstrate the efficiency and effectiveness of the proposed lung nodule detection method, which achieved 96.9% classification accuracy.

## 5. Conclusion

As an important development direction of the Internet of things, the medical Internet of things has been highly valued by the country. Therefore, the intelligent health management of the medical Internet of things has also become a hot issue in many universities and scientific research institutions. Lung cancer screening is an effective way to find early cancer for asymptomatic lung cancer patients. People use the computer to assist and develop CAD system to detect and classify lung nodules. The detection and recognition process of pulmonary nodules is mainly divided into three stages: CT image preprocessing, suspected nodule extraction, and true and false nodule recognition. Preprocessing mainly generates a cube area with annotation information to obtain lung nodule file. Then, the CT image is denoised, and the denoised image is normalized and cut into a fixed-size cube. Suspected pulmonary nodules were extracted mainly using the 3DVNet network and finally using the 3D VGG network to classify true and false nodules. Based on the medical Internet of things, this paper studies the collection of clinical trial data and intelligent differential diagnosis of pulmonary nodules.

In the lung nodule detection model, two data enhancement methods, the lung nodule generation algorithm using three-dimensional pixel statistics and the generation of a countermeasure network, are proposed. The volume-based 3DVNet image segmentation method is used to optimize the training process using a user-defined accuracy function. It is concluded that the data generated by the data enhancement method in this paper can improve the accuracy of the 3DVNet image segmentation network, and the lung nodule generation algorithm based on three-dimensional pixel statistics has the highest accuracy. By introducing the embedding mechanism into the training network model, the 3DVNet image segmentation network, the accuracy of the model can be improved. In the two groups of experiments, the performance of the model in the test set is very poor, and the generalization ability of the model is limited. In the subsequent work, a large amount of data will be used for training to test the performance of the model.

## Data Availability

The data used to support the findings of this study are available from the corresponding author upon request.
